# A novel reverse two-hybrid method for the identification of missense mutations that disrupt protein–protein binding

**DOI:** 10.1038/s41598-020-77992-1

**Published:** 2020-12-03

**Authors:** Olivier Vincent, Angel Gutierrez-Nogués, Adrían Trejo-Herrero, María-Angeles Navas

**Affiliations:** 1 Instituto de Investigaciones Biomédicas Alberto Sols CSIC-UAM, 28029 Madrid, Spain; 2 Departamento de Bioquímica y Biología Molecular, Facultad de Medicina, Universidad Complutense de Madrid, Madrid, Spain

**Keywords:** Biological techniques, Molecular biology

## Abstract

The reverse two-hybrid system is a powerful method to select mutations that disrupt the interaction between two proteins and therefore to identify the residues involved in this interaction. However, the usefulness of this technique has been limited by its relative complexity when compared to the classical two-hybrid system, since an additional selection step is required to eliminate the high background of uninformative truncation mutants. We have developed a new method that combines the classical and reverse two-hybrid systems to select loss-of-binding missense mutations in a single step. The strategy used to select against truncation mutants is based on the two-hybrid interaction between a C-terminal fusion peptide and the Tsg101 protein. We have applied this method to identify mutations in human glucokinase (GK) that disrupt glucokinase regulatory protein (GKRP) binding. Our results indicate that this method is very efficient and eliminates all the truncation mutants and false positives. The mutated residues identified in GK are involved in the GKRP binding interface or in stabilizing the super-open conformation of GK that binds GKRP. This technique offers an improvement over existing methods in terms of speed, efficiency and simplicity and can be used to study any detectable protein interaction in the two-hybrid system.

## Introduction

The yeast two-hybrid system^1^ is a genetic method for detecting protein–protein interactions. This technique is based on the activation of a reporter gene, such as *HIS3*, by a transcription factor formed by association of two interacting proteins, fused to either a DNA-binding or a transcriptional activation domain. The two-hybrid system has been adapted to high throughput detection, which enabled the characterization of the interactome of numerous species, such as human^2^.

The reverse two-hybrid system is based on the classical two-hybrid system^3–5^. This methodology allows the selection of randomly generated mutations that disrupt a given two-hybrid interaction and therefore facilitates the identification of the amino acids involved in such interaction. The detection of the specific residues involved in protein–protein interactions is necessary to determine the physiological relevance as well as the molecular details of these interactions. This technique is based on the use of counter-selection reporters, such as *URA3*. *URA3* inhibits yeast growth in presence of 5-fluoroorotic acid (5-FoA) and mutations that block the two-hybrid interaction prevent *URA3* expression and permit growth in presence of 5-FoA. Although the reverse two-hybrid system was a promising tool for functional studies, it has not been as successful as its predecessor. This difference is due to the fact that most of the interaction-defective mutants selected with the original reverse two-hybrid system are uninformative truncation mutants, and there is also a high background of false positives^5–7^. Different strategies based on the use of a detectable C-terminal fusion have been proposed to discard the non-sense or frameshift mutations and select only the missense mutations. Fusions to the green fluorescent protein have been described^8^ but the additional fluorescence selection step increases the complexity of the method. Fusions to a reporter gene such as *URA3* allow positive selection^9^ but as for GFP, the mutated protein has to be expressed as a triple fusion between the transcriptional activation domain and *URA3*, which could interfere with the two-hybrid interaction. The same limitation exists in the One- plus Two-hybrid System that uses a C-terminal fusion to the Gal4 DNA binding domain (GBD)^10^. To overcome this limitation, C-terminal fusion to the kanamycin resistance gene has been used to discard truncation mutants in *E. coli* prior to the reverse two-hybrid selection in yeast, but this method is not as straightforward and also generates many false positives^7^. Another strategy to minimize the potential interference of a C-terminal fusion protein is to use a short epitope to select full length proteins by immunoblot detection^6^ but this approach is also time consuming. Thus, there is still a need for a rapid and efficient reverse two-hybrid method that selects only missense mutations and eliminates all the false positives.

In this paper, we describe a one-step selection method, the reverse double two-hybrid system (RD2H), which permits the rapid and efficient detection of missense mutations that disrupt protein–protein interactions. This new method combines a reverse two-hybrid selection of interaction-defective mutants and a classical two-hybrid system to select against truncated proteins by using a C-terminal fusion peptide that binds the Tsg101 protein. Mutations are generated by random PCR mutagenesis and Gap Repair recombination, and loss-of-interaction and missense mutations are simultaneously selected with two positive selection markers. The use of a C-terminal short peptide minimizes potential interference of a C-terminal fusion protein, and the simultaneous positive selection efficiently eliminates the truncated mutants and false positives.

In a first application of this method, we identified specific amino acid residues in human glucokinase (GK) required for binding to the glucokinase regulatory protein (GKRP). GK acts as a glucose sensor for the regulation of blood sugar and mutations in *GCK* gene cause maturity onset diabetes of the young, type 2 (MODY2). GKRP binds to the inactive super-open conformation of GK to act as a competitive inhibitor of glucose and to mediate GK nuclear retention in hepatocytes^11^. The GK-GKRP complex has been extensively studied because it is a potential target for antidiabetic therapies and drugs that disrupt GK-GKRP binding produce antidiabetic effect in rodent models of diabetes^12^. The structure of the GK-GKRP complex has also been determined^13,14^ and the identification of GK residues involved in GKRP binding has been undertaken in previous studies^15–19^. However, a systematic isolation of mutations in GK that disrupt its interaction with GKRP has not been addressed. Using the reverse double two-hybrid method, we were able to identify residues within the interaction interface or likely involved in maintaining the structural integrity of the super-open conformation of glucokinase that binds GKRP.

## Results

### The reverse double two-hybrid method

**Figure 1:**
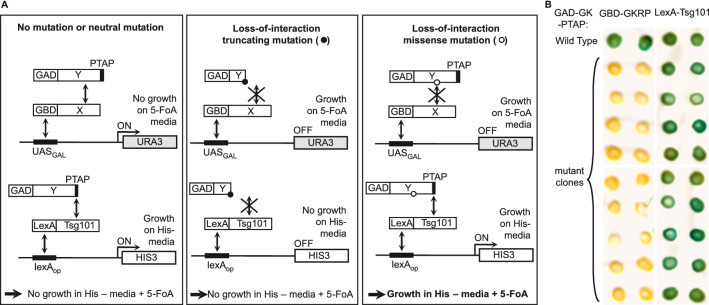
The reverse double two-hybrid strategy. (**A**) Two bait proteins (X and Tsg101) are expressed as Gal4 binding domain (GBD) and LexA fusion, respectively, whereas the prey protein (Y) is expressed as a triple fusion between the Gal4 activation domain (GAD) and a triple repeat of the PTAP motif. GBD-X and GAD-Y-PTAP are expressed from plasmids pGBKT7-X and pACT2-Y-PTAP, while the LexA-Tsg101 construct is integrated in the genome of the yeast strain. Left panel: The interaction between X and Y activates the transcription of the UAS_GAL_-driven *URA3* reporter, which converts 5-FoA into a toxic compound, while the PTAP-mediated interaction between the Y fusion protein and Tsg101 activate the lexA_op_-driven *HIS3* reporter. Therefore, these cells can grow in the absence of histidine but are sensitive to 5-FoA. Middle panel: Truncating mutations in Y that prevent binding to X confer resistance to 5-FoA but also disrupt the PTAP-mediated interaction with Tsg101 and thus prevent growth in the absence of histidine. Right panel: Missense mutations in Y that prevent binding to X do not disrupt the PTAP-mediated interaction with Tsg101 and thus allow growth both in the presence of 5-FoA and in the absence of histidine. (**B**) Selection of missense mutations in glucokinase (Y protein is GK) that prevent its interaction with the glucokinase regulatory protein (X protein is GKRP). GK mutations were generated by PCR random mutagenesis and in vivo gap repair. Colonies growing on histidine-free medium with 5-FoA were selected and plasmids expressing mutated GAD-GK-PTAP were purified and tested for two-hybrid interaction with GBD-GKRP or LexA-Tsg101 in strains carrying a *UAS*_*GAL*_*-* (Y187) or *lexA*_*op*_*-* (CTY10-5d) driven *lacZ* reporter, respectively. ß-Galactosidase lift filter assays are shown for nine representative mutant clones.

The reverse two-hybrid system is a powerful method to identify interaction-defective mutants but its widespread use has been limited by the relative complexity of this technique, which requires several selection steps to eliminate the high background of uninformative truncation mutants. We developed a new experimental strategy, the reverse double two-hybrid system (RD2H), which circumvents these shortcomings. In this new method, we simultaneously select the interaction-defective mutants and eliminate the truncating mutations by using a combination of the reverse and classical two hybrid systems. Reverse two-hybrid selection of interaction-defective mutants is carried out with Gal4 binding domain (GBD) and activation domain (GAD) fusion proteins and the *UAS*_*GAL*_*-URA3* counter-selection reporter containing the upstream activating sequence (UAS) of the *GAL1-10* promoter (Fig. 1A, left panel). We developed a new vector to express the GAD fusion protein as a triple fusion with GAD at the N-terminus and a short peptide containing 3 repeats of the PTAP motif at the C-terminus. This motif present in the HIV-1 Gag protein interacts with the human Tsg101 protein^20^ and we found that a triple repeat of this motif naturally occurring in some HIV-1 strains (GenBank ACS76886.1) results in a much stronger binding to the Tsg101 ubiquitin E2 variant (UEV) domain (data not shown). The PTAP-mediated interaction between the GAD fusion protein and a fusion of Tsg101 to the LexA DNA binding domain activates the positive selection reporter *LexAop-HIS3* (Fig. 1A, left panel) and this second two-hybrid selection is used to eliminate the truncated mutants that do not contain the PTAP peptide and therefore cannot activate *HIS3* transcription. We constructed a new strain, OVY216 that contains the chromosomally integrated constructs *UAS*_*GAL*_*-URA3*, *LexAop-HIS3* and *ADH1p-LexA-Tsg101*.

The strategy used in the RD2H system to identify missense mutations in the protein Y that disrupt its interaction with the protein X is described in Fig. 1A. The strain OVY216 is transformed with two vectors expressing the GBD-X and mutated GAD-Y-PTAP fusion proteins. Random mutations in Y are introduced by mutagenic PCR and gap repair recombination. The interaction between X and Y mediates the association of GAD-Y-PTAP and GBD-X, which induces *URA3* expression and therefore prevents growth in the presence of 5-FoA (Fig. 1A, left panel). The PTAP-mediated association of GAD-Y-PTAP and LexA-Tsg101 activates the *HIS3* reporter and confers growth in the absence of histidine. Loss of X–Y interaction due to missense or truncating mutations in Y prevent *URA3* transcription and permit growth on 5-FoA media (Fig. 1A, middle and right panels). However, truncated mutants lack the PTAP peptide and cannot interact with LexA-Tsg101, so they cannot grow in the absence of histidine (Fig. 1A, middle panel). Therefore, transformants carrying a missense mutation in Y that disrupts its interaction with X are the only ones capable of growing on 5-FoA media lacking histidine (Fig. 1A, right panel). Consequently, missense mutations in Y that disrupt binding to X can be selected in a single step by using random PCR mutagenesis and gap repair method and a combination of two positive selection reporters.

### Screening of GK mutants defective for interaction with GKRP

In a first application of the RD2H system, we aimed to identify mutations in human glucokinase (GK) that disrupt its interaction with the glucokinase regulatory protein (GKRP). We previously showed that GBD-GKRP interacts with GAD-GK in the two-hybrid system^21^. We first cloned the *GCK* coding sequence without stop codon in pACT2-PTAP, between the Gal4 activation domain and PTAP peptide and in frame with both sequences. We then checked the two-hybrid interaction between GBD-GKRP and GAD-GK-PTAP in strain Y187 carrying the *UAS*_*GAL*_*-LacZ* reporter by β-galactosidase filter assay. Blue color was detected (Fig. 1B, left upper row) indicating that the C-terminally fused PTAP peptide does not alter the GK-GKRP two-hybrid interaction. The PTAP-mediated interaction between LexA-Tsg101 and GAD-GK-PTAP was also tested in strain CTY10.5d carrying the *lexAop-LacZ* reporter (Fig. 1B, right upper row).

Random mutations were introduced in pACT2-GK-PTAP by mutagenic PCR and gap repair recombination. To this end, a PCR product containing the GK-PTAP sequence was generated using pACT2-GK-PTAP as template and primers annealing 150–200 bp upstream and downstream of the GK-PTAP sequence. We used either the Mutazyme II or the Taq DNA polymerase under standard conditions to lower the mutation frequency. To increase the transformation efficiency, the OVY216 strain was first transformed with pGBKT7-GKRP and the resulting transformant was co-transformed with the linearized pACT2 vector and the mutagenic PCR product to facilitate cloning by gap-repair of randomly mutated pACT2-GK-PTAP plasmid. Transformants were simultaneously selected for Ura- and His+ phenotypes and the total number of clones was calculated by plating 1% of the cell suspension to unsupplemented media. Ura- His+ transformants are expected to contain mutations in GAD-GK-PTAP that abolish GBD-GKRP binding and therefore prevent *URA3* expression, but do not block the PTAP-mediated interaction with LexA-Tsg101 that activates *HIS3* transcription (Fig. 1A, right panel). We used 3-AT, an inhibitor of His3, to reduce the *HIS3* reporter activity. Although the background growth of his- transformants was not fully suppressed by 1 mM 3-AT, we found that it is completely inhibited by 5 mM 3-AT.

The ratio of positive transformants to the total number of clones screened was approximately 1.25% (500/40,000) and 0.125% (73/58,000) for the Mutazyme- and Taq-based random mutagenesis, respectively. pACT2-GK-PTAP plasmid was recovered from 10 and 9 large colonies from each screen and transformed back into two-hybrid strains Y187 and CTY10-5d to test the interaction of mutant GAD-GK-PTAP with either GBD-GKRP or LexA-Tsg101 by ß-Galactosidase filter assays. In all cases, the interaction between GAD-GK-PTAP and GBD-GKRP was lost while the PTAP-mediated interaction with LexA-Tsg101 was still detected (9 representative mutant clones are shown in Fig. 1B). These results indicate that all the isolated plasmids contain mutations that prevent GK-GKRP binding and do not truncate the GK protein, which validates the screening strategy.

Sequencing of the 19 mutant clones revealed that each contained at least one missense mutation and, as expected, we did not find any nonsense or frameshift mutation that would truncate the protein. *GCK* open reading frame is about 1.4 Kb and the number of missense *GCK* mutations in each clone ranged between one and three for Mutazyme mutagenesis (average of 1.5 missense mutation/kb) or one and two with Taq (average of 1 missense mutation/kb). Analysis of 5 additional clones from the Taq mutagenesis by sequencing of the PCR product amplified from purified yeast DNA led to the identification of mutations previously recovered in the first nine clones. Overall, the number of missense mutations identified more than once was higher with Taq (35%) than with Mutazyme (20%). Because some of these mutants had more than one missense mutation, single mutations were introduced by site-directed mutagenesis into the pACT2-GK plasmid, which does not contain the C-terminal PTAP peptide, and the lack of interaction with GKRP was tested in the two-hybrid system.

**Figure 2:**
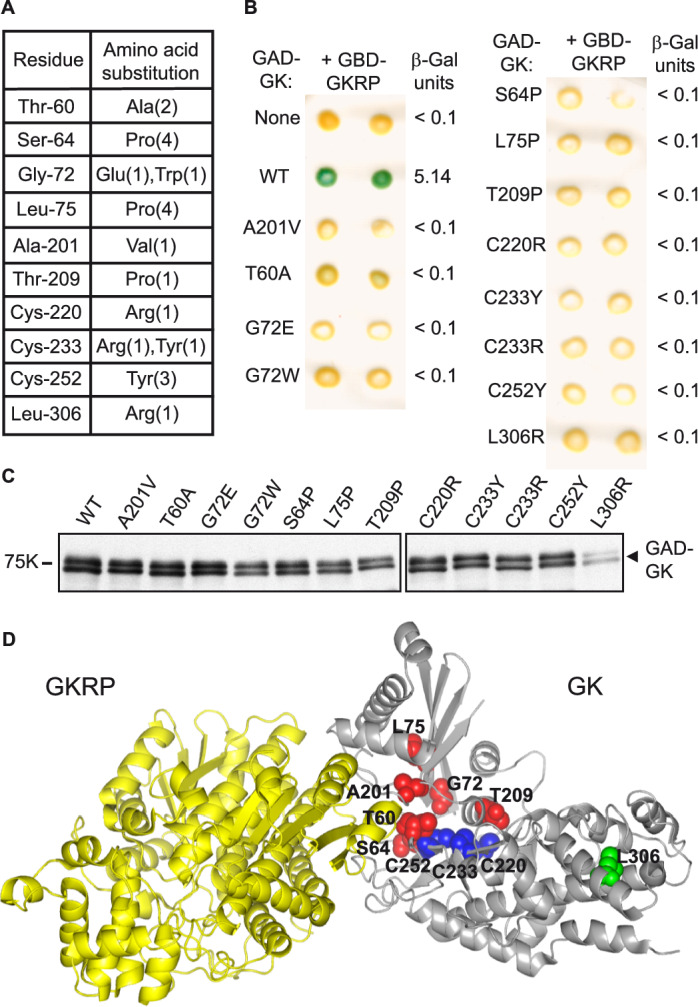
Mutations identified in GK that disrupt its interaction with GKRP. (**A**) The table lists the mutated residues and the corresponding amino acid substitutions identified in the reverse two-hybrid screen. Numbers in parenthesis indicate the number of times the mutation was identified. (**B**) GAD-GK fusions containing the indicated single missense mutations were tested for two-hybrid interaction with GBD-GKRP in strain Y187. Interactions were revealed by β-galactosidase lift filter assays and the β-galactosidase activity is shown on the right. (**C**) Protein extracts from the same transformants were analyzed on an anti-HA immunoblot to visualize HA-tagged GAD-GK fusion. Full-length blots are presented in Supplementary Figure 1. (**D**) Localization of the mutations on the 3D structure of the GK-GKRP complex. Mutated residues near the GK-GKRP interface are highlighted in red, residues in the cysteine ring are colored in blue, and the distant Leu306 residue is displayed in green.

Figure 2A shows the 10 mutated residues in GK identified in this RD2H screen. Some of the mutations were recovered multiple times and different aminoacids substitutions were found for some residues. All the substitutions abolished the two-hybrid interaction between GK and GKRP in β-galactosidase filter and liquid assays (Fig. 2B) and, as expected from our screening strategy, none of them caused protein instability except L306R, which results in a lower level of the GAD fusion protein (Fig. 2C). The position of the mutated residues in the 3D structure of the GK-GKRP complex is shown in Fig. 2D. The main group of residues is localized at or near the GK-GKRP binding interface. Another group of 3 cysteine residues is located in the active site cleft of GK. Finally, the Leu306 residue is present in the large domain of GK, distant from the GK-GKRP interface.

### Effect of the T60A and A201V mutations on GKRP-mediated inhibition and nuclear translocation of GK

**Figure 3:**
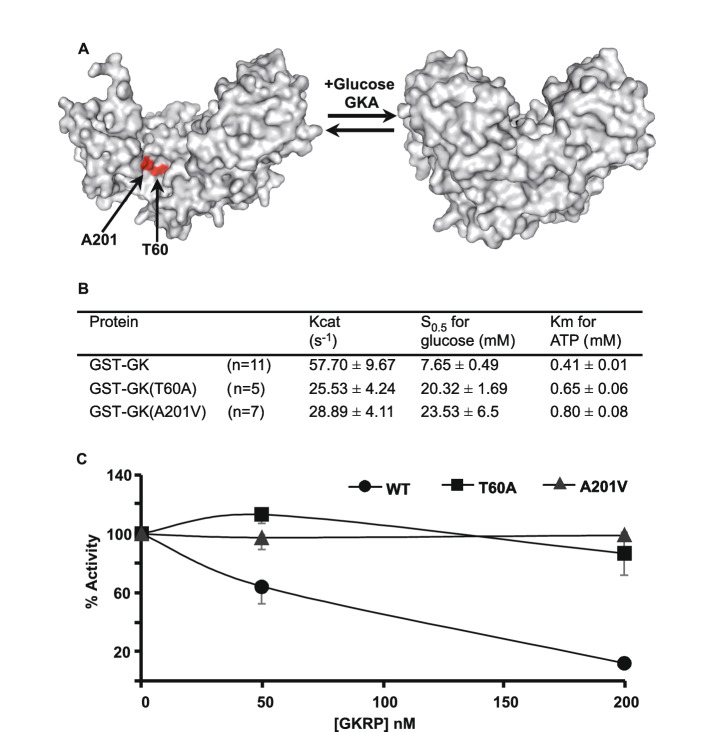
Inhibition by GKRP of wild type and mutant GK activity. (**A**) Surface accessibility of the Thr60 and Ala201 residues on the 3D structure of GK in super-open (left) and closed (right) conformation. (**B**) Enzyme kinetic constants of wild type and mutant GK are shown as mean ± SD for n independent measurements. (**C**) GKRP inhibition assays of wild type and mutant GK. Enzymatic activity was measured with 61 nM of the GST-GK fusion and the indicated concentration of Flag-GKRP.

Out of the ten mutated residues identified in our screen, we focused on two of them, Thr60 and Ala201, which are located at the GK-GKRP binding interface. Strikingly, these two residues are in close proximity and exposed in the GKRP-bound inactive super-open conformation of GK, while they are distant from each other and hidden in the glucose-bound active closed state of the enzyme (Fig. 3A). This observation supports the idea that Thr60 and Ala201 are involved in the GKRP binding site and that conformation-dependent accessibility of these two residues provide the basis for the glucose-dependent regulation of GKRP binding. With the aim of validating the results of our screen and further characterizing these two mutations, we analyzed their effect on the GKRP-mediated inhibition of GK enzymatic activity in vitro. Wild type and mutant GK were expressed in bacteria as Glutathione S-transferase (GST) fusion proteins and purified. We found that both T60A and A201V mutations lower the glucokinase activity by decreasing catalytic constant (Kcat) and increasing affinity constants for glucose (S0.5) and for ATP (Km) (Fig. 3B). Then, the activity of wild type and mutant GK was assayed in the presence of increasing concentrations of purified recombinant human GKRP. In agreement with the loss of GK-GKRP interaction in two-hybrid assays, we found that both mutations abolished the GKRP inhibitory effect on GK enzymatic activity in vitro (Fig. 3C).

**Figure 4:**
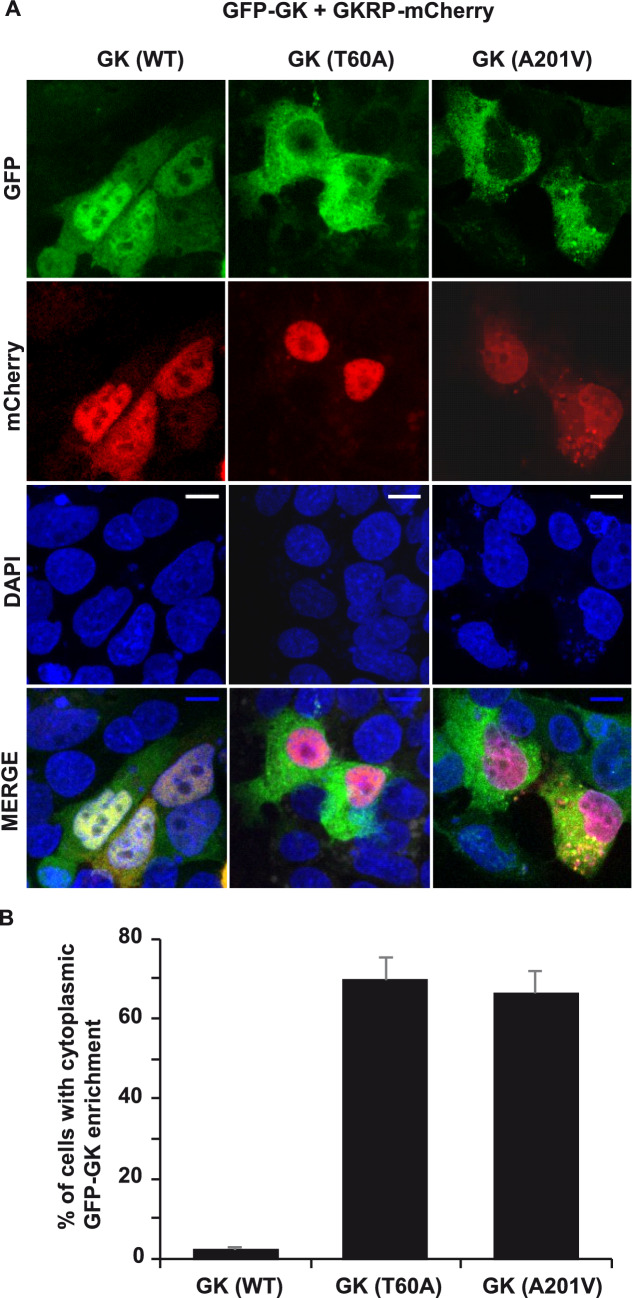
Subcellular localization of wild type and mutant GK in the presence of GKRP. (**A**) Representative fluorescence images of cells cotransfected with GFP-GK variants and GKRP-mCherry. GFP, mCherry and DAPI fluorescence channels are shown. Scale bar 10 μm. (**B**) Percentage of cells with GFP nuclear enrichment.

The translocation of GK to the cell nucleus depends on its interaction with GKRP. Therefore, we analyzed whether the T60A and A201V mutations prevent the nuclear localization of GK. HepG2 cells were co-transfected with plasmids expressing GFP-GK and GKRP-mCherry fusion proteins and analyzed by confocal microscopy. In agreement with previous work^22^, wild type GFP-GK accumulates in the nucleus together with GKRP-mCherry (Fig. 4A). In contrast, the T60A and A201V GFP-GK mutants were enriched in the cytoplasm and nuclear excluded (Fig. 4A,B). Taken together, our in vitro and in vivo data confirm the lack of interaction between mutant GK and GKRP and therefore validate the result of the RD2H screen.

### Functional analysis of the GK mutations T60I and A201P found in MODY2 patients

**Figure 5:**
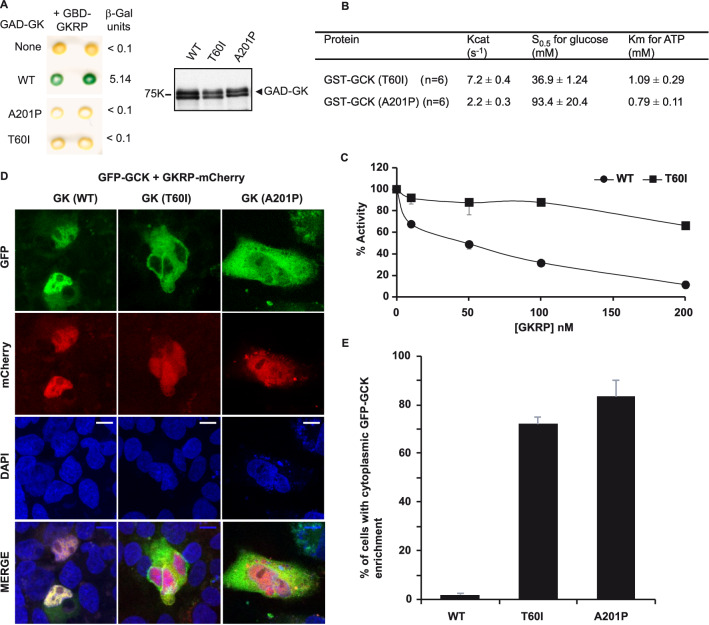
Characterization of the GK mutations T60I and A201P causing MODY2. (**A**) Two-hybrid assay as in Fig. 2B between GBD fusion to GKRP and GAD fusions to GK variants. Shown on right is a western blot analysis of the same transformants, as in Fig. 2C. (**B**) Enzyme kinetic constants of mutant GK as in Fig. 3B (**C**) GKRP inhibition assays of wild type and mutant GK as in Fig. 3C. (**D**) Analysis of the subcellular localization of wild type and mutant GK in the presence of GKRP as in Fig. 4A. (**E**) Percentage of cells with GFP nuclear enrichment.

Other mutations affecting the residues Thr60 and Ala201 in GK (T60I and A201P) have been identified in patients with maturity-onset diabetes of the young type 2 (MODY2) and have not been functionally characterized to date^23,24^. Our aim was to investigate whether these disease-associated mutations also prevent the interaction of GK with GKRP. We first tested their effect in the two-hybrid system and found that both T60I and A201P abolished GK-GKRP binding (Fig. 5A). Enzymatic assays showed that these mutations also lower GK activity, with the strongest effect being observed for A201P, which reduced Kcat by more than 85% and increased S0.5 by more than 12 times (Fig. 5B). When tested in the presence of GKRP, the enzymatic activity of the T60I mutant was largely insensitive to inhibition by the regulatory protein (Fig. 5C). We excluded the A201P mutant from this assay due to its low specific activity. Finally, we analyzed the effect of these two mutations on the GKRP-dependent nuclear import of GK. As shown in Fig. 5D,E, GFP-GK fusions with the T60I or A201P mutations were mainly cytosolic and nuclear excluded in HepG2 cells co-transfected with GKRP-mCherry. Altogether, these results indicate that the two MODY2 mutations T60I and A201P impair both the enzymatic activity of glucokinase and its regulation by GKRP.

## Discussion

The identification of mutations that specifically prevent a given protein–protein interaction is necessary to investigate the physiological significance as well as the molecular basis of this interaction. We developed a genetic method that enables rapid and efficient identification of missense mutations that disrupt protein–protein binding. This method combines the reverse and classical two-hybrid systems, and we named it the reverse double two-hybrid system (RD2H). In a first application of this method, we selected missense mutations in GK that disrupt its interaction with GKRP. We identified ten mutated residues in GK that are associated with a complete loss of GKRP binding. Our results are consistent with previous studies showing that three of these mutations (S64P, G72E and C252Y) led to a loss of inhibition of GK activity by GKRP in vitro^19,25^ and we previously found that the L306R mutation disrupt the two-hybrid interaction with GKRP^22^. Two other mutated residues (T60A and A201V) are located at the GK-GKRP binding interface and we further showed that these mutations result in a complete loss of inhibition of GK activity by GKRP in vitro and the inability of GKRP to promote GK nuclear retention in cell cultures. The involvement of Thr60 and Ala201 in the GKRP binding site is consistent with hydrophobic contacts of the neighbor residues Leu58, Pro59, Tyr61, Val199 and Val200 with GKRP in the super-open GK state^14^. Ala201 and Thr60 become surface-exposed and accessible to GKRP in the super-open GK state, in agreement with the location of Ala201, Tyr61 and Val62 in a hydrophobic pocket involved in locking the closed GK state^26^. Altogether, these findings support the idea that the conformation-dependent accessibility of the Ala201 and Thr60 residues mediates the glucose-dependent regulation of GK-GKRP binding. We further showed that disease-associated mutations of these two residues previously identified in MODY2 subjects also result in a loss of GKRP binding, which could contribute to the clinical phenotype of these patients. Among the other mutated residues identified in our screen, Gly72 likely plays a structural role since it forms a hydrogen bond with Tyr215, which would stabilize the super-open conformation of GK interacting with GKRP^18,27^. Three other mutated residues, Ser64, Leu75 and Thr209, are located in α-helix or β-strands containing or adjacent to the Thr60, Gly72 and Ala201 residues described above. Thus, the inhibitory effect of S64P, L75P and T209P substitutions on GKRP binding may be indirect and due to the proline-induced disruption of these secondary structures. Interestingly, Cys220, Cys233 and Cys252 are part of a ring of 5 cysteine residues in the open GK state, which could form disulfide bridges^28^. The identification of 3 of these 5 residues in our screen strongly suggests that these cysteine bridges stabilize the super-open GK state that binds GKRP. Leu306 is the only mutated residue located far from the GK-GKRP binding region. This residue is part of the GK nuclear export sequence (NES) and we previously reported that the L306R substitution identified in our screen prevents GKRP binding, most likely due to protein folding defects^22^. In line with this, we found that the protein expression level of this mutant is significantly decreased compared to wild type and other mutants. Therefore, we suggest that besides the selection, the protein level of the pray protein should be analyzed to discard false-positives due to protein degradation. Finally, some mutations such as L58R, N204Y and L309R, previously reported to disrupt GK-GKRP binding^17^, have not been identified in our screen, suggesting that it did not reach saturation. In agreement with our results, these mutations are located near the T60A, A201V and L306R mutations identified in this work.

Overall, our results show that the RD2H method effectively eliminates uninformative truncation mutations, as none of the 21 selected mutants contained nonsense or frameshift mutations. Selection against truncation mutants in alternative reverse two-hybrid methods is based on detectable C-terminal fusions such as GFP, *URA3* or GBD. All these strategies have the disadvantage of using protein fusions at both the N-terminal and C-terminal ends. In the RD2H method, the mutated protein is C-terminally fused to a short peptide to minimize the potential interference of this fusion on the two-hybrid interaction. A second advantage of this new technique over previously reported methods is the use of two positive selection markers to simultaneously select the interaction-defective mutants and discard the truncating mutations. The combination of this dual reporter system with random PCR mutagenesis and gap repair in yeast allows the generation and selection of loss-of-interaction missense mutations in a single step, which greatly reduces the time of the procedure. In addition, this strategy efficiently eliminates the background of false positives obtained with other reverse two-hybrid methods. False positives may arise due to the lack of expression of the bait or prey constructs. Pretransformation of the yeast strain with the bait plasmid may be important to achieve a high expression level of the GBD fusion, while the dual reporter system put additional selective pressure on the GAD fusion.

Another important aspect of the reverse two-hybrid system is the mutagenic PCR protocol used to avoid the incorporation of more than one single amino acid substitution in each clone, which depends on both the mutation rate and the length of the PCR product. If a clone contains more than one missense mutation, single point mutants need to be constructed by site-directed mutagenesis in order to identify which mutation is responsible for the loss of interaction, which is time consuming. Due to the relatively large size of the *GCK* ORF (1.4 Kb), we used either Mutazyme II polymerase and low mutation frequency conditions, or Taq polymerase under standard conditions. In agreement with previous work^29,30^, Taq polymerase under standard conditions gives better results, with an average of one missense mutation per Kb. However, the number of mutations identified more than once is higher than with Mutazyme II, probably due to the strong mutational bias of Taq polymerase. Alternatively, two-hybrid mapping could be performed to narrow the binding region in the prey protein and thus reduce the length of the mutagenic PCR fragment.

In summary, we have developed a new reverse two-hybrid method called RD2H, which provide a rapid and highly efficient way to select and identify aminoacid substitutions that disrupt a given protein–protein interaction. The analysis of the GK-GKRP complex with the RD2H method provided new insights into the molecular mechanism linking GKRP binding and GK conformational dynamics. We anticipate that the application of this straightforward technique combined with 3D structural information will be very useful for the structural modeling of protein complexes that have not yet been experimentally characterized.

## Material and methods

### Yeast strains and genetic methods

Yeast strain OVY216 for reverse two-hybrid screening was obtained by crossing the strains MAV203 (*MATα, leu2-3,112, trp1-901, his3-∆200, gal4∆, gal80∆, can1R, cyh2R, LYS2::GAL1-HIS3, GAL1-lacZ, SPAL10::URA3*) (invitrogen) and TAT7 (L40-ura3) (*MATa, leu2-3,112, trp1-901, his3-∆200, ade2-101, gal80∆, LYS2:(lexAop)4-HIS3, ura3:(lexAop)8-lacZ*)^31^. A segregant was selected for the following genotype (*MATa ade2-101, his3-∆200, leu2-3,112, trp1-901, gal4∆, gal80∆, LYS2:(lexAop)4-HIS3, SPAL10::URA3, GAL1-lacZ*) and an integrative plasmid encoding LexA-Tsg101 under the control of the ADH1 promotor (see bellow) was introduced in the *ADE2* locus, giving rise to the yeast strain OVY216 (*MATa ade2-101, his3-∆200, leu2-3,112, trp1-901, gal4∆, gal80∆, LYS2:(lexAop)4-HIS3, SPAL10::URA3, GAL1-lacZ ADE2::LexA-TSG101*). Note that the OVY216 strain carries the *GAL1-lacZ* reporter gene that could be used to confirm the loss of two-hybrid interaction between GBD and GAD fusions. CTY10.5d (*Mata* *ade2-101 his3-Δ200 leu2-Δ1 trp1-Δ901gal4 gal80 URA3::lexAop-lacZ*)^32^ and Y187 (*MATα ura3-52 his3-200 ade2-101 trp1-901 leu2-112 gal4Δ, met15Δ, gal80Δ, URA3::GAL1*_*UAS*_*-GAL1*_*TATA*_*-lacZ, MEL1*)^33^ were used for classical two-hybrid assays. Standard genetic methods were followed, and yeast cultures were grown in yeast extract/peptone/adenine/dextrose (YPAD) or synthetic dextrose (SD) medium lacking appropriate supplements to maintain selection for plasmids^34^.

### Plasmids

Plasmid pACT2-GK-PTAP encoding GAD-GK-PTAP was constructed by cloning the human β-cell *GCK* coding sequence in the BamH1 site of pACT2-PTAP. pACT2-PTAP was generated by inserting a sequence encoding a triple repeat of the PTAP motif (PEPTAPPEPTAPPEPTAPPAE) in the Xho1 site of pACT2 (Clontech) and in frame with the GAD sequence. 2 µm high-copy-number and integrative plasmids encoding LexA-Tsg101 were constructed by cloning the Tsg101 coding sequence in the BamH1 site of LexA(1-202)+PL^35^ or a pRS402^36^ derivative containing the LexA sequence under the control of the *ADH1* promotor and terminator. pACT2-GK and pGBKT7-GKRP encoding GAD-GK and GBD-GKRP have been described previously^37^, as well as plasmids expressing GST-GK, GFP-GK, GKRP-Flag and GKRP-mCherry^22^. Single mutations in pACT2-GK were introduced by site-directed mutagenesis.

### β-Galactosidase assays

Two hybrid interactions were detected by filter lift assays with 5-bromo-4-chloro-3-indolyl-β-d-galactopyranoside (X-gal) as described previously^38^. β-galactosidase was quantitatively assayed in permeabilized yeast cells grown to mid-log phase in a selective SD medium and expressed in Miller units^39^.

### Mutagenic PCR

Random mutagenesis of the *GCK* coding sequence was performed using pACT2-GK-PTAP as template and either the Mutazyme II DNA polymerase from the Genemorph II PCR mutagenesis kit (Stratagene), using the supplier's suggested conditions to achieve low mutation frequency of 0–4.5 mutations/kb (20 rounds of PCR consisting of 95 °C for 30 s, 55 °C for 30 s, and 72 °C for 2 min), or the relatively low-fidelity Taq polymerase with 30 rounds of PCR (94 °C for 30 s, 55 °C for 30 s, and 72 °C for 2 min) and standard conditions. The primers used for mutagenic PCR were OV621 5′-CACTGTCACCTGGTTGGACGG-3′ and OV622 5′-CTATAGATCAGAGGTTACATGGC-3′, which anneal 213 bp upstream and 174 bp downstream of the *GCK* sequence. The resulting PCR product contained the GK-PTAP fusion flanked by 150–200 nucleotides identical to the gapped vector pACT2 linearized with BamH1. Note that the gapped vector does not contain the C-terminal PTAP peptide, which is included in the mutagenic PCR fragment in order to avoid the possible generation of false positives due to vector recircularization.

### Reverse two-hybrid screening

The introduction of random GK mutations in pACT2-GK-PTAP was achieved by gap repair. The OVY216 strain was first transformed with the bait construct pGBKT7-GKRP by the lithium acetate method^40^. The resulting transformant was then co-transformed with the gapped vector pACT2 and the mutagenic PCR products generated by Taq or Mutazyme II DNA polymerase and containing the GK-PTAP sequence. Following transformation, yeast cells were grown in YPAD for an additional 2.5 h before being plated on SD medium lacking adenine, tryptophan, leucine and histidine, and supplemented with 0.005% uracil, 0.1% 5-FOA and 1–5 mM 3-amino-1,2,4-triazol (3-AT). The number of clones screened was calculated by plating 1% of the cell suspension on SD medium lacking adenine, tryptophan and leucine. After a 5-day incubation, we selected the 10 and 9 largest colonies obtained from the Mutazyme- and Taq-based mutagenesis, respectively. Transformants were grown in liquid medium without leucine and mutated pACT2-GK-PTAP plasmids were rescued by yeast DNA extraction and transformation in *E. coli*. These plasmids were co-transformed with either pGBKT7-GKRP in the Y187 strain or LexA(1-202)+PL-TSG101 in the CTY10-5d strain to check by β-galactosidase assays that the two-hybrid interaction between mutated GK-PTAP and GKRP is lost while the PTAP-mediated interaction with Tsg101 is still detected, meaning that it is not a truncating mutation. Mutations were then identified by DNA sequencing. Beside the 19 selected colonies, we analyzed 5 additional transformants obtained from the Taq-based mutagenesis, by direct PCR amplification of the *GCK* sequence from purified yeast DNA and DNA sequencing.

### Immunoblot analysis

Protein extracts from yeast cells grown until mid log phase in selective SD medium were prepared by the NaOH/TCA lysis method^41^ and analyzed by 7.5% SDS-PAGE and immunoblotting with monoclonal antibody against hemagglutinin (HA) (16B12; Covance). Blots were developed with ECL reagent (GE Healthcare, Piscataway, NJ).

### GK enzymatic assays and structural analysis

Recombinant wild-type and mutant GST-GK, and Flag-tagged human GKRP were bacterially expressed and purified as described previously^21,22^. Determination of GK kinetic parameters and GKRP inhibition assays were performed as reported earlier^22^. GK mutations were localized in the crystal structure of human GK in the closed (PDB 1V4S) or super-open (PDB 1V4T) conformation or bound to rat GKRP (PDB 4LC9)^14^ and visualized using the Pymol Molecular Graphics System (Schrödinger).

### Cell culture, transient transfection and fluorescence microscopy

HepG2 cells (ATCC HB-8065, Manassas, VA, USA) were cultured and transfected with plasmids encoding GFP-GK and GKRP-mCherry as described previously^22^, Fluorescence microscopy of transfected cells was performed as reported earlier^22^ with a laser confocal microscope Leica SP2 AOBS equipped with a HCX PL APO 63°x/1.4–0.6 Oil Lbd BL objective, and standard filter sets for visualizing 4′,6-diamidino-2-phenylindole (DAPI), GFP and mCherry. Images were processed with the ImageJ 1.48v software (National Institutes of Health, Bethesda, USA).

### Statistical analysis

For ß-Galactosidase lift filter assays, eight independent transformants were tested of which two representative are shown. The average β-galactosidase activity was calculated from 4 independent transformants and the standard deviation was < 16%. For glucokinase enzymatic assays, enzyme kinetic constants represent mean ± SD for at least 5 measurements from at least 2 independent enzyme preparations. Data for GKRP inhibition assays represent means ± SD for 4 assays using at least 2 independent enzyme purifications. Fluorescence microscopy data represent means ± SD of at least 300 cells visualized in, at least, 3 independent transfections.

## Supplementary information

Supplementary Figure 1.
