# Supplementary material for: A novel reverse two-hybrid method for the identification of missense mutations that disrupt protein–protein binding
Source: Sci Rep. 2020 Dec 3;10:21043. doi: 10.1038/s41598-020-77992-1 (PMC7713115; doi:10.1038/s41598-020-77992-1)
Supplement: Supplementary file 1 — Supplementary Figure 1. [file 41598_2020_77992_MOESM1_ESM.pdf]

Supplementary Material

To

A novel reverse two-hybrid method for the identification of missense mutations that disrupt protein-protein binding

Olivier Vincent, Angel Gutierrez-Nogués, Adrían Trejo Herrero, and María-Angeles Navas

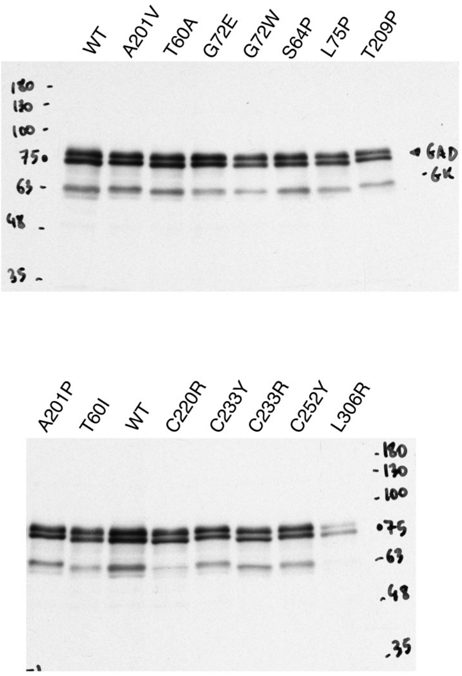

Supplementary figure 1. Full blots for Figure 2C and 5A
